# RETRACTION: Effects of Photodynamic Therapy in Patients With Infected Skin Ulcers: A Meta‐Analysis

**DOI:** 10.1111/iwj.70583

**Published:** 2025-04-18

## Abstract

**RETRACTION:** GuR. , FeiS. , LiuZ. , X. LiuX., Fang, WuH. , ZhangX. , XuG. , and XuF. , “Effects of Photodynamic Therapy in Patients With Infected Skin Ulcers: A Meta‐Analysis,” International Wound Journal 21, no. 3 (2024): e14747, 10.1111/iwj.14747.38445778 PMC10915826

The above article, published online on 06 March 2024, in Wiley Online Library (http://onlinelibrary.wiley.com/), has been retracted by agreement between the journal Editor in Chief, Professor Keith Harding; and John Wiley & Sons Ltd. Following an investigation by the publisher, all parties have concluded that this article was accepted solely on the basis of a compromised peer review process. In addition, the investigation found significant textual overlap between this article and another article by different authors [1]. The editors have therefore decided to retract the article. The authors did not respond to our notice regarding the retraction.
